# Trajectory analysis of the work and life experience of healthcare workers during the COVID-19 pandemic: a longitudinal qualitative study

**DOI:** 10.1186/s12912-023-01520-5

**Published:** 2023-09-27

**Authors:** Pingting Zhu, Meiyan Qian, Amanda Lee, Mark Hayter, Wen Wang, Guanghui Shi, Qiwei Wu, Qiaoying Ji, Xinyue Gu, Hui Zhang, Yinwen Ding

**Affiliations:** 1 School of Nursing, School of Public Health, Yangzhou University, Yangzhou, China; 2 Jiangsu Key Laboratory of Zoonosis, Yangzhou, China; 3 Manchester Metropolitan University, Manchester, England

**Keywords:** COVID-19, Experience, Healthcare workers, Longitudinal study, Nurses, Qualitative research, Trajectory analysis

## Abstract

**Background:**

The COVID-19 pandemic has posed a global health threat and has had a profoundly negative impact on the work and lives of healthcare workers. However, few people know how their experiences have evolved over time.

**Aims:**

To describe healthcare workers’ experiences during clinical responses to COVID-19 and how they changed over time.

**Design:**

A longitudinal qualitative study.

**Methods:**

We undertook a series of four semi-structured qualitative interviews of 14 healthcare workers called as 1st responders to the COVID-19 pandemic. Participants were recruited through purposive snowball sampling. Interviews were undertaken between May 2020 and May 2022 and trajectory approach was used to reveal individual experiences over time. This paper follows the COREQ (Consolidated criteria for Reporting Qualitative Research) guidance.

**Results:**

Data analysis yielded the following four themes: (1) Changes in emotions; (2) Changes in organization and management of care; (3) Changes in knowledge and capabilities; and (4) Changes in outlook on life and career.

**Conclusion:**

Healthcare workers have become stronger in the pandemic and have demonstrated a high degree of professional loyalty and responsibility. However, there is a need to focus on the issue of jealousy and create a harmonious and safe work environment to reduce harm to healthcare workers. Additionally, human resource management strategies must support well-being of healthcare workers and maximize the efficiency of human resource utilization to enable them to respond to current and future needs and emergencies.

**Supplementary Information:**

The online version contains supplementary material available at 10.1186/s12912-023-01520-5.

## Introduction

On December 31, 2019, the city of Wuhan, Hubei Province, China, first reported viral pneumonia of unknown etiology to the World Health Organization, and patient diagnoses soared in a very short timeframe [1]. In response, the Chinese government immediately recruited a variety of healthcare workers (HCWs) (e.g. doctors, nurses, public health professionals, laboratory technicians, health technicians) across China as first responders to COVID-19, and within 3 months, the epidemic in Wuhan was under control [2]. During this period, the virus was discovered across the globe, posing significant threat to global health.

## Background

Globally, healthcare workers have played a significant role in the prevention, control, isolation, and containment of infection [3]. However, they have suffered many challenges. At the start of the pandemic, HCWs had limited knowledge of COVID-19, they lacked prerequisite knowledge, skills and experience of this new virus. Consequently, they reported anxiety and fear (of biological infection), stress, fatigue, sleep disturbances, and many other physical and mental health issues [4–6]. This was exacerbated by professional issues such as staffing and resource shortages, the difficulties posed in seeking, wearing and using Personal Protective equipment (PPE), and intense workloads, so they were at high risk for burnout, traumatic stress, and moral distress [7–9].

Initially, many countries adopted strict measures such as home quarantine, social lockdown, and school suspension to restrict the movement of people and the spread of the virus, but the virus evolved into different variants, meaning each country had to adjust their COVID-19 related policies [10]. The initial response ‘zero COVID policies’ and lockdown measures have evolved over the past three years, with mitigation strategies for viral coexistence (with the exception of China) [11]. Repeated global outbreaks have increased pressure on China’s epidemic prevention and control measures, with sporadic and repeated outbreaks noted in some areas [12]. HCWs were required to respond to each outbreak for COVID-19-related tasks. In August 2021, Yangzhou, Jiangsu Province experienced an epidemic culmination, the government immediately organized designated infectious disease hospitals, and citywide testing to control spread, with a large number of HCWs participating in this battle [13].

Typically, healthcare workers’ experiences with health and the health care system change over time. Recent studies reveal that HCWs previously exposed to COVID-19 experienced symptoms consistent with post-traumatic stress disorder, with decreased motivation to work, fear of re-exposure to the disease, and even desire to quit their profession [14, 15]. Other studies reveal increased professional identity and sense of responsibility [16, 17]. When re-engaged in COVID-19 care, some HCWs showed more experience and confidence, but some still had difficulty adapting [18]. To date, most studies of HCWs’ COVID-19-related experiences are based on cross-sectional studies, with focus on initial response at the outbreak, meaning there is limited knowledge of long-term influences in HCW first responders. Although a previous longitudinal qualitative study investigated the impact of the pandemic on the psychosocial and emotional well-being of nurses in the UK, it was only conducted in the year following the start of the pandemic [19]. HCWs experienced a second outbreak in a short period of time and became exhausted due to the lack of containment strategies, however, the experience of Chinese HCWs may be different. In addition, this study focused more on the analysis of content at a single point in time, however when the research interest is an experience or process, it is more appropriate to analyze it in a way that emphasizes individual trajectories [20].

Therefore, this study presents a novel approach, using a series of longitudinal narratives of the first responder HCWs (nurses and doctors). The aim is to explore experiences of HCWs during their clinical response to COVID-19. This approach enables an improved insight into the impact of COVID-19 on HCWs. It offers a novel approach to highlight lessons learned, to underpin effective physical and mental health safeguarding measures for HCWs, and to guide future clinical practice in pandemic response.

## Methods

### Aim

To describe healthcare workers’ experiences during clinical responses to COVID-19 and how they changed over time.

### Study design

We performed four round interviews across a two-year period of HCW first responders in a longitudinal qualitative study to explore their experiences during the mission against COVID-19. QLR (qualitative longitudinal research) is an “evolving methodology” which offers rich and helpful exploration and in-depth understanding of the dynamic evolution of people’s lives – to reveal changes over time [21]. The COREQ (Consolidated criteria for Reporting Qualitative Research) was used to ensure we provided sufficient methodological detail and analysis for rigor and credibility [22].

### Sampling and recruitment

Purposive and snowball sampling yielded HCW participants from three grade ‘A’ tertiary hospitals in Yangzhou, Jiangsu Province who were transferred to Wuhan as first wave responders to pandemic and were the first wave HCWs worldwide to be involved in treating patients with COVID-19. This methodology enabled us to reveal the small population of HCWs who were in the initial call to pandemic response, and then, through snowballing, to recruit their colleagues. Inclusion criteria included (1) age ≥ 18 years; (2) involvement in frontline rescue missions for at least 4 weeks; (3) proficiency with electronic communication devices (mobile phone, tablet and computer); (4) informed consent and voluntary participation in being interviewed; (5) intent to remain in the role as HCW for duration of the study. All interviews were conducted online to reduce contact between researchers and participants, in line with containment measures. Fourteen participants were recruited to the first round of interviews. The sample was determined as complete when we identified no new topics at initial interview. As a longitudinal study, we used the same population sample for all three consecutive interviews and there was no attrition during the course of this research and all participants remained in their allocated roles throughout the study [23].

### Data collection

We conducted four semi-structured interviews with the participants from May 2020 to May 2022 through telephone and online communication (Wechat software). Interviews were held at initial support period in Wuhan (T0), six months (T1), one year (T3) and two years (T4) when they returned to Jiangsu.

The interview questions were formulated by the researchers (PT Z, MY Q, QW W) according to the research purpose and based on relevant literatures [6, 24]. Two experts in nursing and public health research were consulted in the development of the questions (MH, AL). Then we piloted these questions with a small number of healthcare workers (n = 3) and further modified them to form the final interview guide. The main core questions included: (1) Can you tell me how your experience of caring for patients with COVID-19 is different than before?; (2)Can you tell me how your life and work have changed during this time?; (3)Can you tell me what has changed in your mind, mood, behavior and body during this time? Supplementary 1 illustrates the specific semi structured questions used to inform interviews. The first interview focused on understanding HCW work experiences and psychological influences of responding to COVID-19, and interviews undertaken at T2, 3 and 4 were further used to explore any work or life changes which participants revealed at follow up.

Three independent interviewers (PZ; QW; MQ) were experienced qualitative researchers who were interested in the experiences of healthcare workers during COVID-19 and had extensive experience in conducting COVID-19 related interviews. PZ has a PhD, QW and MQ has an MNS, they were female and independent of the clinical setting. A total of three data collection researchers were allocated participants to interview across all four time points to ensure continuity. Interviews were conducted at a time considered convenient for participants, who were also working clinically. Prior to the interview, all participants were informed about the purpose and procedures of the research and were asked to provide verbal informed consent. Nobody else was present besides the participants and researchers. We established a trusting relationship with the participants by exchanging pleasantries, leading with an interview outline, incorporating exploratory questions and encouraging participants to express their own views. The order and content of the interviews were adapted to the specific situation during the interview. The interviews were digitally recorded and transcribed in full with the consent of the participants. Field notes were taken during and after interviews. Anonymized transcription data were held in secure storage facility to maintain confidentiality. The nurses were numbered with the letter N (N1-N10) and the doctors with the letter D (D1-D4) according to the order of the interviews. Interviews duration was 12–95 min.

### Data analysis

We (authors) applied trajectory analysis to perform longitudinal data analysis. In contrast to recurrent cross-sectional analysis, trajectory analysis enables researchers to gain an understanding of the experiences of individual or groups of participants over time. This approach uses sequential time-ordered matrices to initially organize themes according to a point in time, capturing the trajectory of change in related concepts in subsequent analyses and helping to understand what led to what [20]. Based on findings from our data, we developed a thematic framework to assist further data analysis [24].

Two coders (PZ; MQ) repeatedly read the transcribed text to familiarize themselves with the data and independently coded the transcribed text line by line with the help of the qualitative analysis software Nvivo12 [25]. Similar codes were clustered to create sub-themes and themes [26]. Themes derived from the data rather than obtaining them from pre-existing themes in the literature. Once coded, the data was organized into matrices and presented across appropriate cells to construct the final matrices for analysis, we developed one matrix for each unit of analysis. As each transcript was a repeat interview of an individual participant over time, we reviewed the content of the participant’s second interview and recorded any changes that had occurred since T1, including no change, improvement, deterioration, or new concepts, in areas that aligned with the categories in the framework. We repeated the same exercise with the T3 and T4 transcripts for each participant. Once the matrix was completed for each participant, we then began the longitudinal analysis, a step that focused on exploring how the concepts or domains in the matrix changed or remained unchanged over time. To better organize the results, we referred to the literature to organize the 2nd matrix, whose x- and y-axes are organized according to unit analysis and themes respectively [20]. This process was entered manually, without the aid of qualitative analysis software. We compared the longitudinal summary profiles for each unit of analysis to identify similarities and differences in changes in participants’ work and life experiences over time. We resolved the differences between coders through discussion and group consensus.

### Rigor and reflexivity

The rigor of results was assessed in terms of credibility, dependability, confirmability and transferability to develop the trustworthiness of research [27]. Credibility: The data collection researchers disseminated these themes to participants through electronic communication devices for further discussion. This ensured our themed findings offered a true representation of their experiences. Dependability: All authors of this study were experienced in qualitative research and the original research protocol was peer reviewed by an external researcher. Confirmability: Accuracy in the data collection and analysis process was optimized through both field notes and interview transcriptions to avoid or minimize errors or biases. Transferability: The researcher specifically described the geographic location of the study, the demographics of the participants, and the timeframe for data collection and data analysis, allowing these findings or methods to be transferred from one group to another. Immediately following each interview, the researchers wrote notes about personal feelings, biases, and insights, and reflexively discussed through regular meetings with team members, critically considered their personal biases as researchers, and reflected on study procedures.

## Results

The 14 healthcare workers (10 nurses and 4 doctors) came from three hospitals in Jiangsu province, of which 3 were men and 11 were women. The work experience of the participants ranged from 6 to 18 years. Eight of them were from the ICU, emergency department, and respiratory department. During the two-year follow-up period, 2 nurses were married and 3 nurses were promoted from primary nurse to Nurse-in-Charge. (Table 1).

Four themes and twelve sub-themes emerged from the data analysis (Table 2). Results are organized to highlight descriptions of the ways in which healthcare workers experiences evolve or remain constant over time. At the same time, we plotted figures for each theme (Figs. 1, 2, 3 and 4) to more clearly and intuitively show the general changes experienced by most healthcare workers during the interview.

**Table 1:** Characteristics of participants

Healthcare workers ^a^	Gender	Age	Marital Status	Professional Title	Work experience, years	Department	Recruitment Avenue
N1	Female	33–35	Married	Nurse-in-Charge	12–14	General Surgery	Center 1
N2	Female	32–34	Single-Married	Primary nurse- Nurse-in-Charge	8–10	ICU	Center 2
N3	Female	39–41	Married	Nurse-in-Charge	17–19	Traditional Chinese Medicine	Center 3
N4	Female	35–37	Married	Nurse-in-Charge	12–14	Respiratory Department	Center 1
N5	Female	35–37	Married	Nurse-in-Charge	15–17	Respiratory Department	Center 1
N6	Female	28–30	Single	Primary nurse- Nurse-in-Charge	6–8	Traditional Chinese Medicine	Center 2
N7	Female	29–31	Single-Married	Primary nurse - Nurse-in-Charge	7–9	Emergency Department	Center 3
N8	Female	40–42	Married	Associate chief nurse	18–20	Neurosurgery Department	Center 3
N9	Female	38–40	Married	Associate chief nurse	18–20	General Surgery	Center 2
N10	Female	30–32	Married	Nurse-in-Charge	7–9	Neurosurgery Department	Center 3
D1	Male	41–43	Married	Associate chief physician	17–19	Emergency Department	Center 1
D2	Male	40–42	Married	Associate chief physician	17–19	Respiratory Department	Center 2
D3	Male	38–40	Married	Attending physician	14–16	ICU	Center 3
D4	Female	32–34	Married	Attending physician	6–8	Respiratory Department	Center 2

^a^ N: Nurse; D: Doctor

**Table 2:** Themes and Sub-themes

Themes	Sub-themes
Changes in emotions	Negative emotions; Positive emotions
Changes in organization and management of care	Self-protection; Acute goals with care; Teamwork; Human resource challenges
Changes in knowledge and capabilities	COVID-19 knowledge levels; Critical care management capabilities; Communication ability; Grief counseling ability
Change in outlook on life and career	Close relationships with family members; Sense of professional mission

### Changes in emotions

**Fig. 1:**
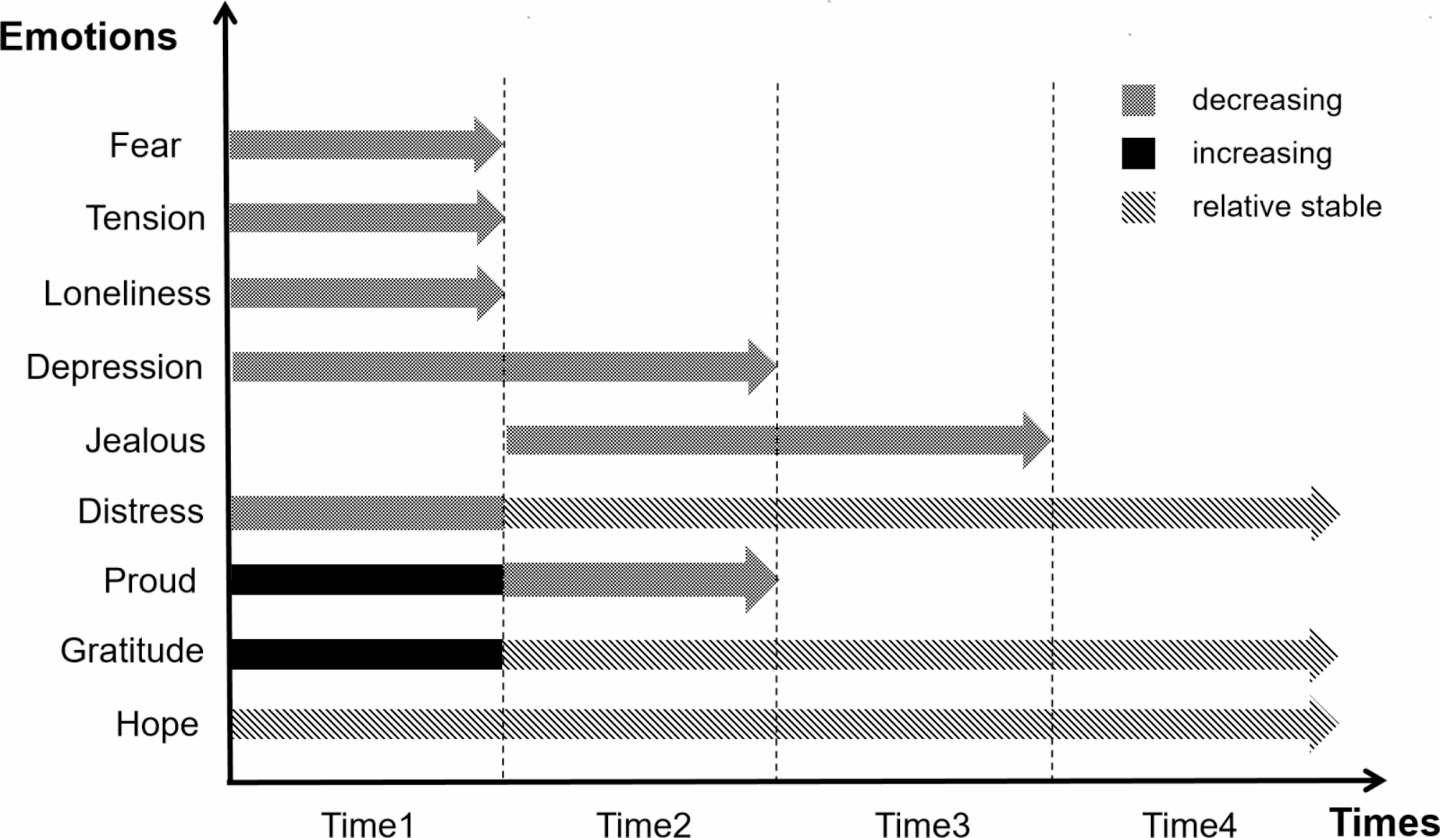
Changes in emotions

#### Negative emotions

All HCWs reported negative emotions, which peaked at T1 and gradually disappeared over time. Fear of infection was cited the most because of increasing numbers of infections and COVID-19 related deaths. This was particularly exacerbated by infection and death of work colleagues. As management and control processes were standardised, most participants reported their fear decreased or disappeared by middle to late stages of T1. When faced with the virus outbreak again (T2, 3 & 4), participants consistently reported they were ‘no longer afraid’, which some attributed to their increased knowledge and experience.

*“It was an annoying and depressing time for me because they (patients) were always vomiting and we were afraid of being infected.“ (N1, T1)*.

*“Just leave that scene, that fear slowly faded away.” (D1, T2)*.

*“(Collecting nucleic acid) was risky because you didn’t know who was carrying the virus… But we’ve all gone through the strict protection training, so it was OK.” (N7, T3)*.

*“I haven’t worried about it, because I think my experience is quite adequate. I didn’t worry too much about that. I think as long as you follow the standards process, you won’t get an infection.“ (N3, T4)*.

The feelings of loneliness, anxiety, tension, and hopelessness experienced by the participants ended with the end of the support mission, yet some participants’ reported depression and mental ill health at T2, with forgetfulness, inability to concentrate, short temper, and sleep disturbances. They reported trying to forget their experiences in Wuhan because recall made them feel miserable and was mentally difficult. These symptoms were relieved by the third interview, with participants even missing the experience.

*“I was devastated, (I) thought, I’ve been here for over fifty days, (why) won’t you let me go back?” (N7, T1)*.

*“(I)felt like I couldn’t breathe well and had a headache, but it wasn’t really a headache. As soon as I mentioned (Wuhan) it slowly started to get stuffy and my head started to hurt and (I) felt sad.“ (N4, T2)*.

*“We had to take the caregiver’s temperature every day, check their escort card every day, check their nucleic acid report, we have added a lot of trivial work.” (N1, T3)*.

*“I missed Wuhan a little bit, which was a valuable experience.“(N2, T3)*.

We found that participants’ distress did not change over time, but for many, the factors influencing distress changed. For example, during the support period in Wuhan, participants mainly faced the great challenge of caring for COVID-19 patients. When they returned from their victory against the epidemic, they were labeled “heroes” and their words and actions were more scrutinized, and mistakes were magnified, but they could not argue with them. At T3, nurses were often assigned to nucleic acid collection duties and strict ward management orders due to the demand for regular management, and they reported a greater workload than before. The last interview had participants indicating that they were more upset psychologically because it was their own city that was sick. Participants have been in varying degrees of distress for a long time.

*“Although I made some small mistakes in life, but it was infinitely magnified… Such words were very hurtful.” (N4, T2)*.

*“As a local medical team, it must be very sad to see all COVID-19 patients were local people. (N7, T4)*

#### Positive emotions

As time passed, the participants’ emotions gradually became more positive. The participants expressed their pride and sense of accomplishment that their expertise and knowledge could be useful during the time of national crisis, which reflected their personal value. Some of the participants’ pride and sense of accomplishment persisted, but some of them faded away. This is mainly because they were all rewarded as well as honored to varying degrees after their victory against the epidemic, yet they were subjected to jealousy and even denigration from those around them. This experience of being envied would continue to affect them, and they will not mention the experience actively, they emphasizing the need to be down-to-earth repeatedly.

*“Some people were very congratulatory and some were jealous… people were cynical… They speculated every day that we got a million dollars in prize money, but we didn’t.” (N8, T2)*.

*“Because after all, others have not experienced it, and when you talked to them, they might think you are showing off.” (D3, T4)*.

Participants remained grateful for the two years of follow-up. The government and hospital provided adequate organizational support and various forms of psychological support at T1, and colleagues and friends provided a lot of guidance and concern, all of which were an effective guarantee for them to devote themselves to their work. China has long adhered to a dynamic zeroing policy, with infection rates and mortality rates greatly reduced. Participants said that they and their families, especially the elderly, were well protected, and that “reunion” is something that Chinese families aspire to. At the same time, participants took on the role and responsibility of their families, and the long-standing understanding and selfless support of their families were the solid backing for the healthcare workers to put in their work, whether they were involved in front-line rescue missions or in nucleic acid testing.

*“My high school classmates cared about me and supported me when they heard I was going to Wuhan, which made me feel touched.” (N10, T1)*.

*“The policy of our country was really quite good, in fact (I) haven’t been working for long, (but) to get such a high honor and such good treatment, I thought omg it was really worth it!” (N8, T2)*.

*“I was thankful to my family, especially my husband, for always being behind me and he took good care of the kids.” (N5, T3)*.

*“Thanks to our national leadership has been adhering to the dynamic zeroing policy… Some of my family members are old with a lot of underlying diseases, and they cannot withstand the infection.” (N2, T4)*.

The end of the epidemic was the constant hope of the participants. Hope as a positive spiritual force has sustained them through all the events that have occurred over the past two years and they have shown a stronger sense of mission and responsibility during their work.

*“(I) hope the epidemic ends quickly, otherwise the country’s human and financial resources are too costly.” (D1, T1)*.

*“I hope the epidemic can end quickly. I haven’t traveled with my family for a long time.” (N1, T3)*.

### Changes in organization and management of care

**Fig. 2:**
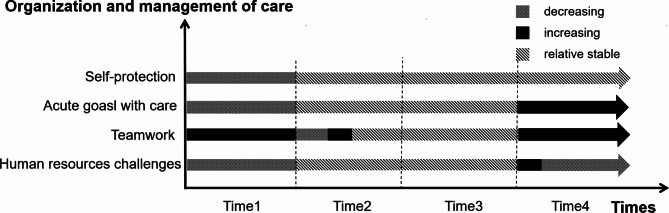
Changes in organization and management of care

#### Self-protection

Participants showed us a gradual normalization of the self-protection process. Early support participants tended to feel fearful and took strict measures to protect themselves, such as wearing three layers of gloves, spending at least half an hour taking off protective clothing, half an hour showering, taking prophylactic herbs, etc., but these measures often made caring for the patient more difficult as well as more strenuous for the individual. When re-engaged in rescue missions, participants said that they were more relaxed in their self-protection than before and realized that they had over-protected themselves before. Also, having suffered from a shortage of protective clothing, masks and other protective supplies at T1, participants paid attention to replenishing supplies at home in their daily lives, just in case.

*“After a period of time, I think the protection is even a little bit too much, but protection is the way to go.” (N9, T1)*.

*“You had to wear a mask when you went out, and also at work, which was the most basic measure (to protect yourself).” (D2, T2)*.

*“(I) stored some masks and disinfectant from time to time, and I also stored more supplies at home, unlike before, used up and bought again.“” (N2, T3)*.

*“Before entering the warehouse everyone must be assessed in putting on and taking off protective clothing, and only those who got a pass could go in… It was already a proven process, just follow the requirements (to complete).” (N9, T4)*.

#### Acute goals with care

The participants’ acute goals with care changed over time. Due to the tight schedule and heavy workload during support in Wuhan, participants often had to be satisfied with reaching the goal of curing patients, and patients’ emotional needs and patients’ rights, such as the right to information and personal safety, were always ignored. When conflicts arose between the interests of patients and the safety of the healthcare workers themselves, participants said that there is no way out but to protect themselves first in order to save more patients. However, when they joined the rescue tasks again, they did their best to improve the quality of care for patients due to their increased experience and competence.

*“I see nothing wrong with protecting ourselves while making sure our normal nursing work have been done.” (N6, T1)*.

*“I will care for them a little bit more. I will go to comfort them and talk to them if they are in a bad mood.” (N1, T4)*.

#### Teamwork

After COVID-19 outbroke in Wuhan, hospitals across the country dispatched healthcare workers to form medical teams. These healthcare workers from different hospitals were unfamiliar with each other and the new environment, and had different work habits and adaptations. Participants said that working with unfamiliar people was a challenge and that there was a lot of coordination and collaboration involved in trying to develop a “fighting force” quickly. As time passed, the participants became better at working with each other. After returning to Yangzhou, they returned to work in their original sections, but the participants said they were not comfortable with their original jobs at first and did not have the same tacit understanding with their colleagues, which was restored as time passed. Familiar companions gave them a sense of security when they were back on the front line.

*“We were all new colleagues and we needed to have a bonding process. Because we were not familiar with each other, we couldn’t have the same level of tacit understanding as did in the past.” (N7, T1)*.

*“At first it was a bit hard to get used to the pace of their (colleagues’) work when I first came back …… Soon (I) got used to it.” (N1, T2)*.

*“(We) were a little bit prepared and we knew each other, which made us feel safe.” (N3, T4)*.

Leaders always played an important role in team management during the two years of the pandemic. They needed to have extensive clinical experience and assign tasks rationally based on the team members’ strengths to make the team work as efficiently as possible. They tried to balance tasks for fairness in work scheduling, and were also responsible for communicating and consulting with people when problems were identified. During regular management period, leaders needed to be more flexible in scheduling and allocating human resources as needed as it has become common to have staff shortages due to arranging for people to help out with nucleic acid collection and vaccinations.

*“My captain was a little more communicative and coordinated the issues I found a little better.” (N5, T1)*.

*“I tried to be fair when I was in charge of scheduling, and I sometimes chose to sacrifice my time off to keep the ward in order.” (N9, T3)*.

#### Human resource challenges

The human resource challenge has been an unavoidable problem for healthcare workers over the past two years. The high number of confirmed COVID-19 cases brought the medical system to the brink of collapse, and the shortage of staff was eased as different batches of healthcare workers went to Wuhan to support them. Simultaneously, with the establishment and improvement of rules and regulations, the scheduling of healthcare workers was more reasonable and the division of labor was clearer. After returning to Yangzhou, the healthcare workers were engaged in regular epidemic prevention and control, which added a lot of work to them. In addition to the daily work in wards and clinics, they were sometimes sent to nucleic acid testing sites, fever clinics and vaccination sites, which meant that fewer people had to undertake more work. During the outbreak in Yangzhou, the government set up nucleic acid testing sites in various communities, hoping to learn about the spread of the epidemic and block the source of infection by conducting multiple nucleic acid tests on a large scale. Some healthcare workers were sent on nucleic acid collection missions, which required them to perform nearly five million tests in three days. The task was undoubtedly stressful. However, due to the sudden outbreak of the epidemic, it was impossible to recruit enough professionals in a short period of time, and the actual number of staff relative to the number of needs was not enough.

*“There were other medical teams coming to support us and to manage the patients together, so we felt less pressure.” (N6, T1)*.

*“It was true that some people resigned, but there were also a lot of people that came in, it was the same as before.” (N8, T2)*.

*“We have to arrange nurses to go to the sports park to vaccinate the residents, this is the hospital, this is required by the health commission.” (N9, T3)*.

*“The biggest problem was the lack of staff. Instead of having four-hour shifts, we actually had six-hour shifts.” (N5, T4)*.

### Changes in knowledge and capabilities

**Fig. 3:**
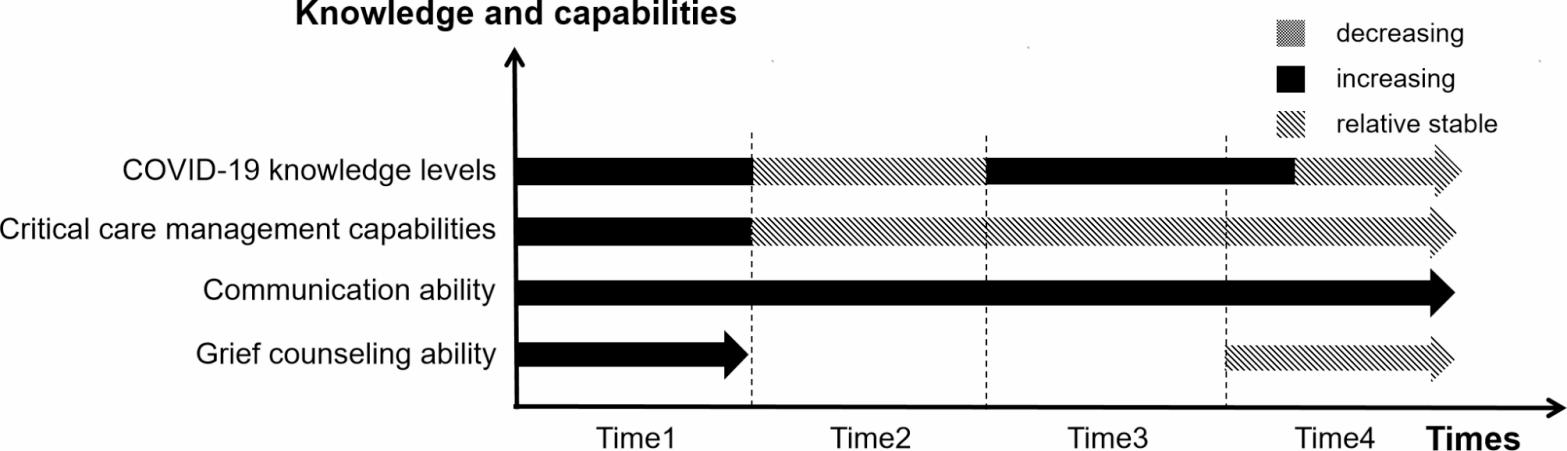
Changes in knowledge and capabilities

#### COVID-19 knowledge levels

As time passed, participants’ knowledge of COVID-19 increased. Participants reported that they had only received brief training before entering the ward and knew very little about the virus, and they had to refer to books and draw on the experience of those who had previously been involved in the treatment of large infectious diseases (e.g., SARS). They feared that they would not be able to bring timely help to the patients, or even cause harm to them. However, after several months of rescue work, their COVID-19 knowledge level was comprehensively improved, including disease care, first aid, protection, disinfection and isolation measures, and final disinfection disposal, etc. This knowledge accumulation provided them with support and confidence when they re-entered the rescue work.

*“We referred to the books for some knowledge and experience of people who have participated in SARS.” (D1, T1)*.

*“I was certainly not afraid, we have encountered these things, just like the normal process to work.” (N6, T4)*.

In the two years that COVID-19 evolved and emerged in different branches, participants said they kept abreast of developments about COVID-19 through the Internet and had constantly updated information.

*“The virus was COVID-19 in Wuhan, but now it is delta, and it seems to be several times more spreadable than the original one in Wuhan.” (N6, T3)*.

#### Critical care management capabilities

Critically ill COVID-19 patients often required multi-organ function support, and providing care for COVID-19 patients was different for most healthcare workers from their previous jobs. These challenged the knowledge and skills of healthcare workers, especially for new or those who lacked experience working in infectious diseases or intensive care units.

They expressed fear and anxiety about working in an intensive care unit and about operating instruments and equipment. However, during the virus outbroke in Yangzhou, they were able to master the use of instruments such as ECMO and hemodialysis machines. They had good specialist skills in ventilator management, airway management, and circulatory support management, and were more confident in treating critically ill patients.

*“I was worried about what would happen if I needed to use these instruments but I didn’t know how to use. It was with this in mind that I had concerns.” (N6, T1)*.

*“(We) also came across (patients) who needed to be rescued, and (we) needed to give them CPR, ventilator or other things, we did our best to save them.” (D3, T3)*.

*“The last patients left were just really sick, some on ECMO, then on a ventilator, and on CRRT, and the patients were particularly sick.” (N4, T4)*.

#### Communication ability

The communication abilities of the participants were enhanced over time. In Wuhan, participants had to resolve cultural and linguistic differences with patients, colleagues had to communicate with each other to enhance cooperation, and leaders were responsible for communicating and coordinating the distribution of supplies and the scheduling of team members. They became more willing to interact with others and acquired more communication skills. Upon returning to their previous jobs, participants also described changes in their relationships with their leaders and colleagues. During the regular management period, the hospitals adopted a strict management system where only one family member was allowed to accompany each patient, and that family member also had to provide proof of a negative nucleic acid test result and could not come and go as they pleased. Many patients and their families often did not understand these policies and argued with the healthcare workers, who had to find ways to explain and calm them down.

*“This experience has given me comradeship and made me more willing to communicate with others, and (I) thought communication was a beautiful thing too.” (N7, T1)*.

*“After returning to Jiangsu, I think the changes I made were that I was more serious in my work and communicated better with my leaders and colleagues.” (N10, T2)*.

*“Some patients and families did not understand and quarreled with us, although I understood their feelings, but that was my job.” (N2,T3)*.

#### Grief counseling ability

Initially, due to the highly contagious nature of the virus, many patients and their family members might all be infected and sent to hospitals for treatment in succession. Unfortunately, not everyone could be cured, some patients received news that their family members had died while they were still in treatment themselves, and the patients suffered from the loss of their loved one. In this case, the participants, as healthcare workers, needed to provide grief counseling to patients to help the bereaved ease their inner pain and get through the grieving period. At T1, participants continued to learn and deepen their understanding of grief counselling and their grief counselling skills improved significantly. When re-engaged in the rescue mission and confronted with patients in need again, participants experienced empathy fatigue despite being able to provide comfort.

*“I would go to comfort her. The distance was very close, I would be a little nervous, and I would try to maintain some distance.” (N3, T1)*.

*“Some patients were so unstable that we had to comfort them.” (N5, T4)*.

### Change in outlook on life and career

**Fig. 4:**
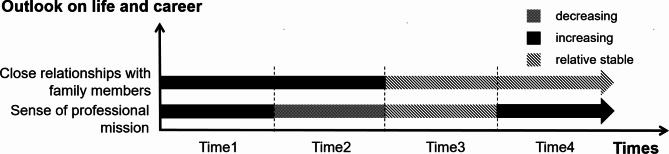
Changes in outlook on life and career

#### Close relationships with family members

In Wuhan, participants were required to work full time and could not go home for several months. Because they were concerned about each other’s health, participants reported that they had more frequent phone or WeChat contact with their families than usual, and that their close relationship with their families had improved during this time. However, they always felt guilty that they could not spend good time with their families due to their busy schedule, whether they were involved in the treatment of COVID-19 patients or in regular management. Therefore, they cherished the time with their families more and were grateful that they were healthy.

*“(My mother and I) usually communicated for about half an hour (every day). (I wanted to) see her, see my mom. (I) missed her and wanted to see how she was doing, how she was doing at home.” (N2, T1)*.

*“The child was closer to me, and he might also be afraid that his mom would be away for a long time again.” (N10, T2)*.

*“I was very busy at work and couldn’t take care of either the elderly or the children.” (N5, T3)*.

*“I want to spend more time with my children, more time with my husband, and hopefully travel if I have the chance.” (N10, T4)*.

#### Sense of professional mission

The participants’ sense of professional mission changed over time. When asked why they chose to support Wuhan, some participants said that it was their job to participate in the frontline of the fight against the epidemic and that they should do a good job, while others said that it was a responsibility and a professional mission to participate in the rescue work. Therefore, despite their own fears, they chose to stay on the front line. Racing against death to save a life, coupled with the gratitude and encouragement received from patients, the participants’ sense of professional mission enhanced. Upon their return to Yangzhou, they were awarded some honors and prizes, but some of the participants suffered envy from colleagues and “blame” from individual patients, which to some extent shook the participants’ sense of professional mission. Over time, however, the fluctuations leveled off. The participants gradually got used to the regular management, which provided a solid guarantee for the health of patients, despite the impact on their work and life. They said that they will not leave the medical profession because they are the guardians of human health. The participants also concluded by saying that they are always ready and will not hesitate to come forward if the country needs them.

*“They (patients) were grateful to us…… Most doctors or healthcare workers were like that, and it was their mission to bring patients back from the brink of death.” (D2, T1)*.

*“Although I did not verbally say that I regretted going to support Wuhan, but I thought in my heart: why should I protect you? … (I was) just sad inside.” (N4, T2)*.

*“You can’t control people’s ideas. All you can do is do yourself, work hard and treat patients well. It’s just your responsibility.” (N4, T3)*.

*“I was always prepared and willing to go if the need arose.” (N8, T4)*.

## Discussion

This study explored the experience of HCWs during their clinical response to COVID-19 through trajectory analysis. Consistent with previous findings, HCWs commonly experienced high levels of negative emotions such as fear, anxiety, and helplessness in the early stages of the pandemic [28]. Prolonged exposure to traumatic and distressing events without adequate psychological preparation could have a cumulative influence on the mental health of HCWs, leading to post-traumatic stress reactions upon their return to former lives [29]. However, we found that these states faded over time, with HCWs demonstrating greater resilience, being able to adapt to changes caused by unexpected events and recover from negative experiences [30]. In addition, we found that negative experiences catalyzed the onset of positive emotions. HCWs expressed gratitude, hope, and persisted over time, which was related to the multidimensional social support they received from friends, patients, colleagues, government, especially family members [31], which could also explain why the psychological trauma of them gradually disappeared.

Similar to several previous studies, HCWs took pride in being able to save patients’ lives and being respected by them [32], but surprisingly, this pride faded due to jealousy from those around them. To thank HCWs for their efforts in the fight against the Wuhan epidemic, the Chinese government has introduced a series of incentives [33], including consolatory grants, title evaluation, rear security, and travel benefits. For HCWs with outstanding work performance, they would be given priority in the evaluation of titles, and the conditions could be relaxed or broken accordingly upon approval, which means they have more chances to be promoted in their work compared to those who were not involved in support. Jealousy arises when a person believes or perceives that another person (a competitor) is threatening his or her own source of material and psychological gain [34]. Research has shown that jealousy is a powerful emotion that can cause a range of hostile or unethical behaviors that endanger the physical and mental health of HCWs [35], thereby reducing the quality of work, affecting interpersonal communication and behavior in organizations, and hindering the development of professional roles [36]. The influence seems to be long-lasting, and many HCWs in our study repeatedly stressed not to share the experience voluntarily, otherwise it would be seen as showing off. Meanwhile, although the experience of being jealous has somewhat reduced HCW’s sense of professional mission, it has not disappeared, which shows their high degree of loyalty and responsibility to the profession.

In contrast to other studies, HCWs in our research showed more confidence and calmness than fear when faced with a new epidemic climax [18, 28]. One possible reason was that over time, uncertainty was replaced by experience and they adapted to the process. Consistent with the results of other studies, HCWs have improved their knowledge and capabilities across the board through repeated clinical practice over time and by actively learning through multiple channels such as the Internet, team members, and social media [37]. Among them, the communication ability of HCWs was in a state of continuous improvement, which indicated that effective communication was crucial to support HCWs through long-term crisis, and could provide HCWs with help in knowledge sharing, emotional support, resource allocation, etc. [38]. However, the critical care management ability of HCWs was relatively stable. The possible reason is that HCWs came from general departments rather than ICU, emergency department and other dangerous departments, and they rarely encountered severe patients in their daily work. In addition, COVID-19 specific protocols and guidelines, involving infection prevention and control, emergency care, healthcare environment management, resource management, and other areas were constantly being revised and improved, which helped to improve efficiency, treatment success, and prevention and control effectiveness [39]. As long as these regulations and guidelines were strictly followed, the infection rate and death rate of HCWs would be significantly reduced, allowing them to shift their efforts from focusing on self-protection to providing higher quality services to patients, thereby reducing the likelihood of experiencing ethical dilemmas.

Human resource was a key link in epidemic prevention and control, but its management was always a challenge for leaders. Studies have shown that pandemics stimulated additional self-awareness in leaders, leading them to rapidly develop new knowledge and skills to seek quick solutions, including arranging for the movement of people, making complex operational decisions, and facilitating teamwork [40]. However, no matter during climax of the epidemic or during the regular management, the huge workload kept the human resources of HCWs under strain, and we needed to think about how to reduce the loss of personnel and make the limited human resources play a greater role on the basis of the inability to recruit more HCWs. Firstly, it is necessary to build a continuously updated and dynamically supervised medical human resources sharing platform to provide scientific and effective reference basis for allocation [41]. Secondly, conduct comprehensive, multi-level, regular and continuous training in public incident rescue and emergency medicine to enhance the knowledge and skills of HCWs and prepare them adequately. In addition, the mental health of HCWs needs continuous attention, providing psychological counseling and guidance to HCWs with problems to ensure a vibrant, professional and healthy medical team. Finally, incentive policies should be developed to give frontline HCWs appropriate honorary recognition, financial support, and leave incentives to improve the morale of them during the epidemic [9].

Although reluctant to be called heroes or angels, HCWs have always stood bravely like heroes in the front line of the fight against the virus, and they have become more aggressive and stronger [42]. They gave new meaning to their lives, some valued harmonious interpersonal relationships and became closer to their families, colleagues, and friends, while others began to re-plan their lives and tried new things [43, 44]. As HCWs, they are willing to stay on the job and fight alongside others.

### Limitations

Firstly, the epidemic prevention required all interviews to be conducted through voice calls, which may affect the integrity of the results, because it is impossible to observe the gestures and expressions of the participants during the interview. Secondly, this study was conducted only for HCWs in Yangzhou, which may have some geographical limitations and restrict the representativeness of the results.

## Conclusions

This study captured the trajectory of HCWs’ experiences over time as they changed or remained unchanged through a longitudinal design. HCWs became stronger and showed greater psychological resilience under the COVID-19 pandemic, which was associated with multidimensional factors such as national policies and social support. Managers need to identify and focus on the presence of jealousy in the clinic and adopt strategies to hinder the negative impact of jealousy on HCW individuals as well as on the development of the healthcare industry. Rational use of human resources is crucial to the fight against COVID-19. Managers should establish a human resource sharing platform, strengthen the education and training, psychological services and implement incentive policies for HCWs. These results can inform strategies for safeguarding the physical and mental health of HCWs and improving the management of COVID-19 and similar public health emergencies in the future.

### Electronic supplementary material

Below is the link to the electronic supplementary material.

Supplementary Material 1

## Data Availability

The datasets used and/or analyzed during the study available from the corresponding author on reasonable request.

## Abbreviations

COREQ: Consolidated criteria for Reporting Qualitative Research
HCWs: Healthcare workers
PPE: Personal protective equipment
UK: United Kingdom
N: Nurse
D: Doctor
ECMO: Extra Corporeal Membrane Oxygenation
CRRT: Continuous Renal Replacement Therapy
