# Supplementary material for: Trajectory analysis of the work and life experience of healthcare workers during the COVID-19 pandemic: a longitudinal qualitative study
Source: BMC Nurs. 2023 Sep 27;22:333. doi: 10.1186/s12912-023-01520-5 (PMC10523727; doi:10.1186/s12912-023-01520-5)
Supplement: Supplementary file 1 — Supplementary Material 1 [file 12912_2023_1520_MOESM1_ESM.docx]

Supplementary 1 Interview guide

| T1（2020.2-2020.5） |
| --- |
| Are there any differences between your original daily work and the work in Wuhan? What are the specific differences? |
| Can you tell me how you feel when treating patients with COVID-19? |
| Did you encounter any challenges during the rescue? How did you overcome them? |
| During the rescue, did your work have any influences in your family life and interpersonal relationship? |
| Have you changed mentally and physically since you arrived in Wuhan? Can you describe your mental, emotional, behavioral and physical changes? |
| Do you have a new perspective on your career as a result of this rescue mission? |
| T2（2020.6-2020.11）、T3（2020.11-2021.4）、T4（（2021.5-2022.4）） |
| Can you tell us something about your life and work during this period? |
| Can you tell me how your life and work have changed during this time? |
| Can you tell me what has changed in your mind, mood, behavior and body during this time? |
| Do you have a new perspective on your career now? |
| Why do you think there has been change or lack of change during this time? |
| Can you tell me how your experience of caring for patients with COVID-19 is different than before? |
| Looking back on the past experience, do you have any feelings? Is there anything else you'd like to say? |
| Can you tell me about the difference between participating in the Yangzhou outbreak response compared to supporting Wuhan? (onlyT4, if applicable) |
